# Detection of circulating tumor cells that predicts the efficacy of neoadjuvant chemotherapy for locally advanced triple-negative breast cancer

**DOI:** 10.3389/fmed.2025.1536971

**Published:** 2025-04-30

**Authors:** Cong Wang, Jingyue Li, Tingli Luo, Shenglin Zhu, Mingda Zhao, Yicai Jia, Yefu Liu

**Affiliations:** Department of Breast Surgery, Liaoning Cancer Hospital and Institute, Dalian Medical University, Shenyang, China

**Keywords:** triple-negative breast cancer (TNBC), circulating tumor cell (CTC), CD133, neoadjuvant chemotherapy (NAC), circulating tumor stem cells (CTSCs)

## Abstract

**Objective:**

This study aims to assess the predictive potential of circulating tumor cells (CTCs) and circulating tumor stem cells (CTSCs) in locally advanced triple-negative breast cancer (TNBC) undergoing neoadjuvant chemotherapy (NAC) compared to the RECIST 1.1 standard.

**Methods:**

We analyzed 112 patients with TNBC at the Liaoning Tumor Hospital. CTCs and CTSCs were evaluated before NAC, on the first NAC cycle day, and after NAC. We assessed the ability of positive CTSCs after the first cycle to predict NAC resistance (requiring regimen change) with a 91% specificity. Additionally, we analyzed CTC dynamics during the first NAC cycle to predict efficacy (often reaching MP4 or MP5) with 87% sensitivity and 80% specificity.

**Results:**

Positive CTSCs post-first cycle predicted NAC resistance with high specificity (91%). The gradual decline in CTCs during the first NAC cycle indicated NAC efficacy, allowing the regimen to continue with a sensitivity of 87% and specificity of 80%.

**Conclusion:**

This study suggests that positive CTSCs after the first NAC cycle predict resistance, thereby facilitating early detection (≥ 6 weeks earlier than RECIST). Gradual CTC reduction during the first cycle predicts efficacy, enabling regimen continuation. CTCs and CTSCs show promise as predictive markers for NAC efficacy in patients with locally advanced TNBC.

## Introduction

Neoadjuvant chemotherapy (NAC) is a standard treatment for patients with locally advanced breast cancer (1). Pathological complete remission (pCR) after NAC can predict a good prognosis for patients with triple-negative breast cancer (TNBC) (2). Previous studies have suggested that even if chemotherapy is resistant, patients still have high inefficiency and poor prognosis (3). The Response Evaluation Criteria in Solid Tumors (RECIST) 1.1 criteria are widely used to evaluate the efficacy of clinical treatment (4). However, they are only applicable under restrictive conditions (clear lesions, adequate tumor size, and at least 2 NAC cycles); Errors will occur when degeneration, necrosis, or fibrous tissue proliferation occurs within the tumor (5, 6). Moreover, RECIST can only evaluate whether the completed NAC is valid and cannot predict whether NAC is valid (7). Therefore, for patients with locally advanced TNBC disease, a clinical indicator that predicts the efficacy of subsequent NAC after the first cycle of NAC is urgently needed.

Circulating tumor cells (CTCs) are tumor cells that fall from tumor tissue and circulate in the blood (8). Multiple studies have shown that CTCs are important markers of malignant tumor metastasis and can predict the chemotherapy response and prognosis of multiple metastatic tumors (9–11). Studies have shown that significantly increased CTCs can be detected 5–6 months before the diagnosis of colorectal cancer recurrence (12). Besides, it has been reported that the CTC count decreased in some patients after NAC while it increased in others (13, 14). Circulating tumor stem cells (CTSCs), derived from cancer stem cell-like cells, have been found in many cancers, including breast cancer, and are thought to contribute to chemotherapy resistance and metastasis (15, 16). CD133 is a cancer stem cell-like cell marker associated with the progression of breast cancer (17). The expression of CTSCs with the CD133 marker has great potential for clinical significance in the prognosis of locally advanced TNBC (18).

Therefore, it is not only necessary to investigate the changes in CTC count and expression levels of CTSCs during NAC but also to determine whether the changes in CTC count and CTSCs can predict NAC response in patients with locally advanced TNBC disease during the first NAC cycle.

## Methods

**Figure 1:**
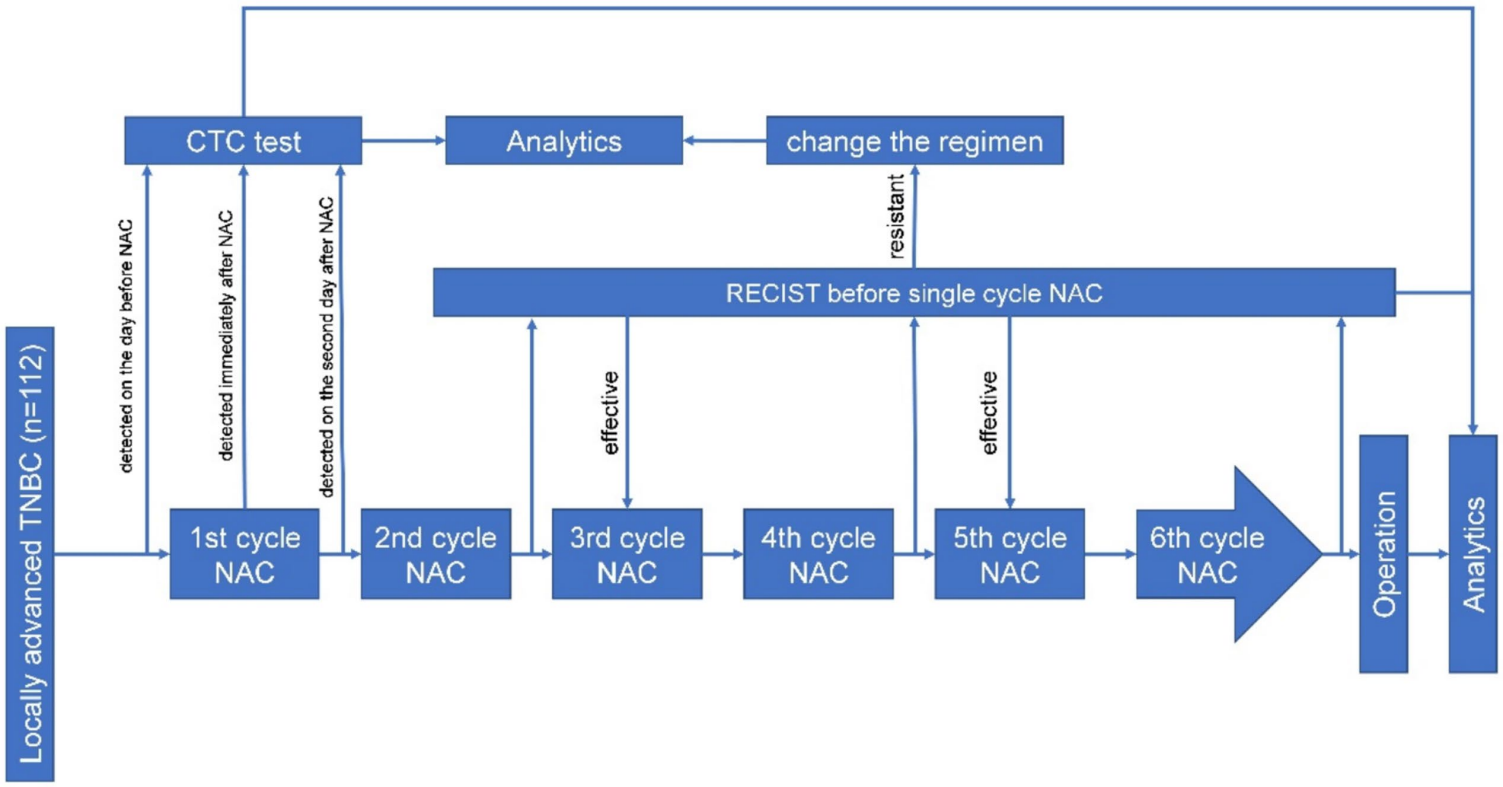
The study flowchart.

Between January 2021 and December 2022, a total of 112 patients with locally advanced TNBC were admitted to the Department of Breast Surgery, Liaoning Cancer Hospital and Institute, Shenyang, China, for needle biopsy, NAC, and surgical treatment. Inclusion criteria: (1) No previous history of breast cancer or other malignant tumors; (2) The diagnosis of invasive ductal carcinoma is confirmed through puncture biopsy; (3) Pre-NAC immunohistochemistry (IHC) showing negative estrogen receptor (ER), progesterone receptor (PR), and human epidermal growth factor receptor 2 (HER2) expression. The exclusion criteria were as follows: (1) Metastatic breast cancer. (2) Pregnancy or lactation; (3) Inability or unwillingness to participate. The detailed process is shown in Figure 1. In this study, the self-control method was used, whereby the patient underwent the CTC test 1 day before, during, and after the first cycle of chemotherapy. Then, the test was conducted according to the neoadjuvant chemotherapy criteria for locally advanced TNBC; that is, the RECIST evaluation was conducted before the first cycle of chemotherapy, and according to the RECIST score, the chemotherapy regimen was changed according to the criteria for the increased stable disease and progressive disease (Figure 1).

### CTCs and CTSCs detection

Cyttel test kits (CS1, CS2, CS3, and CF1 solutions; Cyttel Biosciences Inc., Jiangsu, China) and methods were used to isolate and count CTCs. A total 3.2 mL of peripheral blood samples were collected in BD Vacutainer tubes (Becton, Dickinson and Company, Franklin, NJ) and rinsed once with 1 × CS1 buffer. The samples were then centrifuged at 650 g for 5 min, and the red blood cells were lysed with 1 × CS2. The reaction mixture was again centrifuged at 650 g for 5 min, and the resulting cell pellet was re-suspended in 1 × CS1. Bead-bound leukocytes were placed on top of CS3, a special gradient centrifuge, and isolated at 300 g for 5 min. The resulting solution containing any CTCs was applied to a single slide (Thermo Fisher Scientific, Franklin, MA) using a magnetic scaffold, fixed, and dried for subsequent analysis using the imFISH method. Any CTCs present were fixed on the slide with CF1 and immersed in a 2-fold sodium citrate solution at 37°C for 10 min. According to the manufacturer’s instructions, dehydration was performed using 75, 85, and 100% ethanol for 3 consecutive minutes before adding 10 μL of the CEP8 hybridization solution to the slide. Slide samples were denatured at 76°C for 5 min, hybridized for 1.5 h, immersed in formamide for 15 min, and incubated twice for 5 min with 2-fold sodium citrate saline. Finally, enriched cells were incubated with Alexa Fluor 594–conjugated anti-human CD45 for 1 h and fixed using DAPI-containing medium.

**Figure 2:**
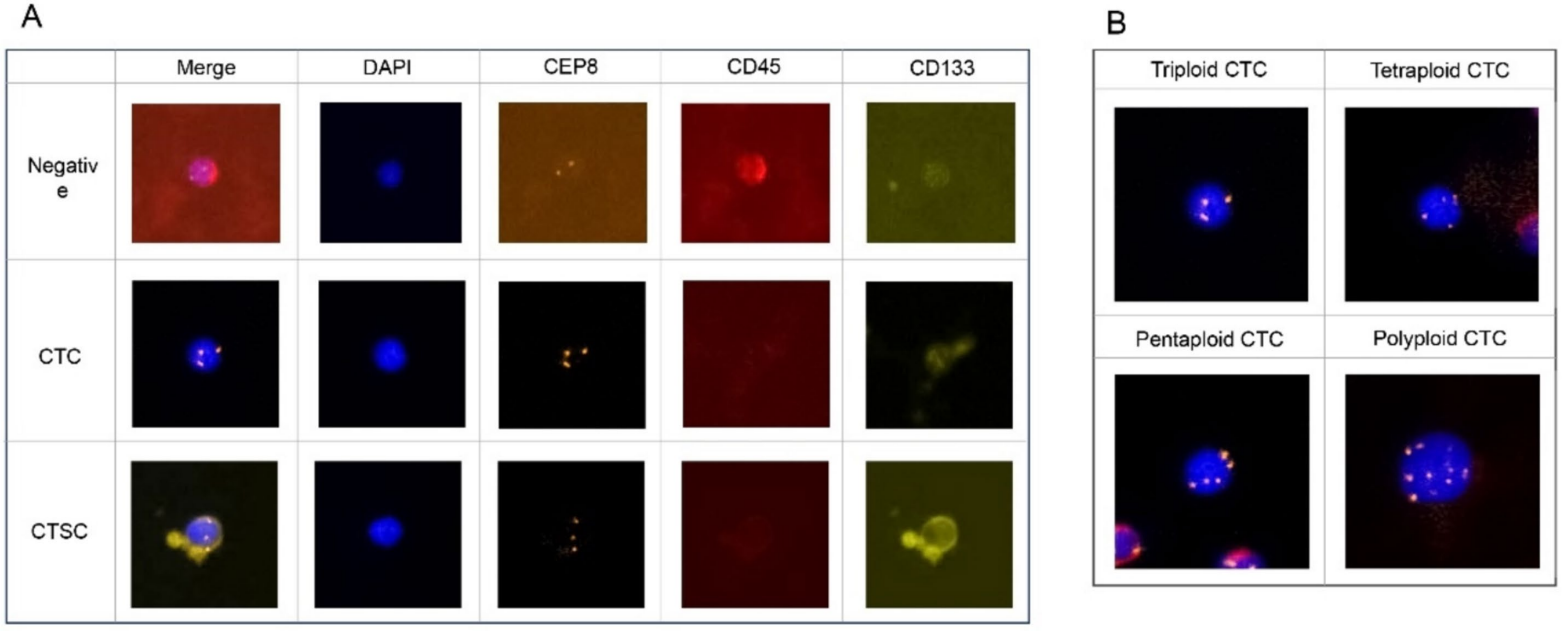
**(A)** CTCs were defined as CD 133-/DAPI+/CD45-/CEP8 + cells, whereas CTSCs were defined as CD133+/DAPI+/CD45-/CEP8 + cells. **(B)** CEP8 + was defined signals having three or more CEP8 signals in a cell.

The expression of CD133 was detected by immunofluorescence. Enriched cells were fixed on a glass slide with CF1, incubated with anti-CD133 antibody for 2 h, and then washed thrice with washing buffer. A fluorescein-conjugated secondary antibody was added, and the slides were incubated at 37°C for 30 min before rinsing thrice with washing buffer. The glass slide was then fixed with DAPI-containing fixing medium and observed under a fluorescence microscope. CTCs were defined as CD 133-/DAPI+/CD45-/CEP8 + cells, whereas CTSCs were defined as CD133+/DAPI+/CD45-/CEP8 + cells (Figure 2A). In addition, CEP8 + was defined as the presence of three or more CEP8 signals in a cell (Figure 2B).

### Statistical analysis

Statistical Package for the Social Sciences (SPSS) software (version 13.0 for Windows; SPSS Inc., Chicago, IL) was used for data analysis. The *χ*^2^ test or *t*-test was used for comparisons between groups, as appropriate. The receiver operating characteristic (ROC) curve and area under the curve (AUC) were used to determine diagnostic performance. A *p* < 0.05, the difference was considered to be statistically significant.

## Results

**Figure 3:**
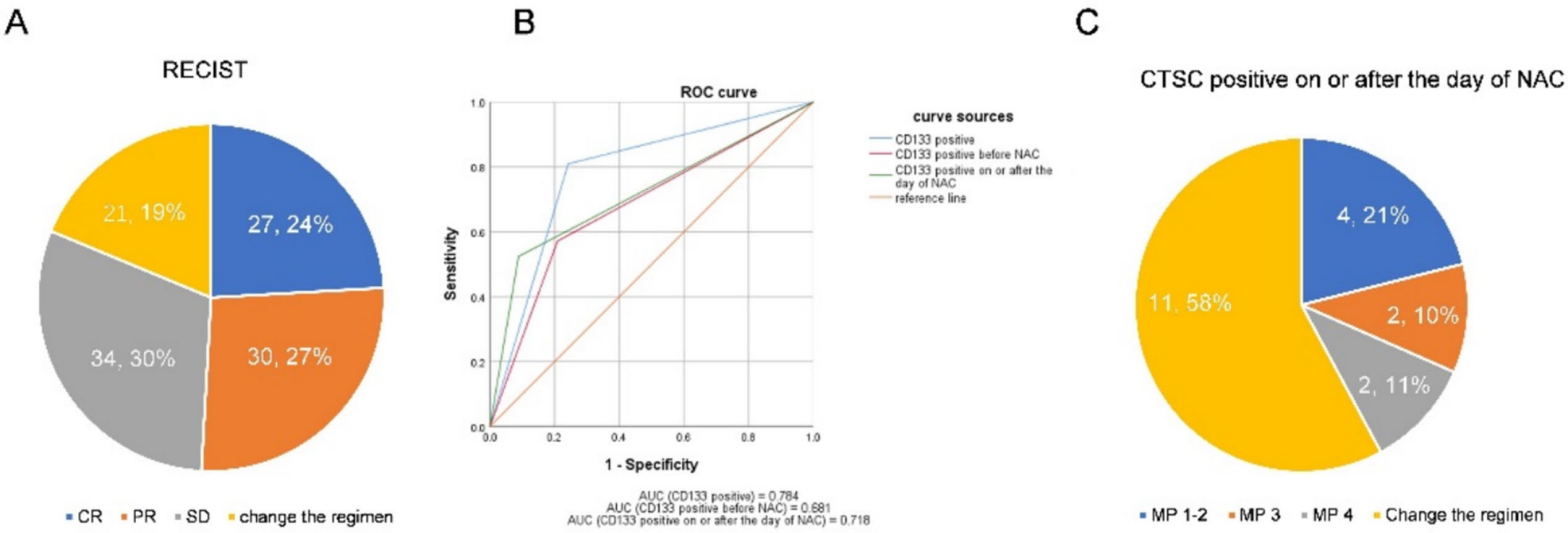
**(A)** Imaging evaluation showed complete remission (CR) in 27 patients (24%), PR in 30 patients (27%), and SD reduction in 34 patients (30%), whereas a change in NAC regimen (increased stable disease or progressive disease) was observed in 21 patients (19%). **(B)** Based on the ROC curve, the AUC positive for CTSCs was 0.784, the AUC positive for CTSCs before NAC was 0.681, and the AUC positive for CTSCs on or after the day of NAC was 0.718, depending on whether or not the chemotherapy regimen was changed. **(C)** A total of 19 patients with CTSCs positive on or after the day of NAC were included in this study, including 11 patients (58%) who changed the protocol.

This study included 112 patients with locally advanced TNBC. Patient characteristics are as shown in Table 1. Imaging evaluation showed complete remission (CR) in 27 patients (24%), PR in 30 patients (27%) and SD reduction in 34 patients (30%), whereas a change in NAC regimen (increased stable disease or progressive disease) was observed in 21 patients (19%) (Figure 3A). Based on the ROC curve, the AUC positive for CTSCs was 0.784, the AUC positive for CTSCs before NAC was 0.681, and the AUC positive for CTSCs on or after the day of NAC was 0.718 (Figure 3B), depending on whether or not the chemotherapy regimen was changed. Sensitivity and specificity are shown in Table 2. A total of 19 patients with CTSCs positive on or after the day of NAC were included in this study, including 11 patients (58%) who changed protocol (Figure 3C).

**Table 1:** Patient characteristics with locally advanced TNBC (*n* = 112).

Characteristics	Levels	Overall
Age		47.71 ± 7.404
Menopause	Premenopausal	63 (56.3%)
	Postmenopausal	49 (43.8%)
Family history	No	98 (87.5%)
	Yes	14 (12.5%)
Tumor size		3.13 ± 1.008
Histological grade	I	8 (7.1%)
	II	17 (15.2%)
	III	87 (77.7%)
Tumor location	Areolar	4 (3.6%)
	Inner upper	8 (7.1%)
	Inner lower	12 (11.6%)
	Outer lower	14 (12.5%)
	Outer upper	73 (65.2%)
Ki-67		68.04 ± 10.296
Chemotherapy	AC-TH/TAC	79 (70.5%)
	TP	12 (10.7%)
	Change the regime	21 (18.8%)
Number of metastasized lymph nodes		6.00 ± 6.254
Number of lymph nodes		24.75 ± 3.947

TNBC, triple-negative breast cancer.

**Table 2:** The sensitivity and specificity with CTSCs positive, CTSCs positive before NAC, CTSCs positive on or after the day of NAC, CTCs with stepped descending.

Condition of CTSCs and CTCs	Sensitivity	Specificity
CTSCs positive	81.0%	75.8%
CTSCs positive before NAC	57.1%	79.1%
CTSCs positive on or after the day of NAC	52.4%	91.2%
CTCs with stepped descending	86.5%	79.5%

CTSCs, circulating tumor stem cells; NAC, neoadjuvant chemotherapy; CTCs, circulating tumor cells.

**Figure 4:**
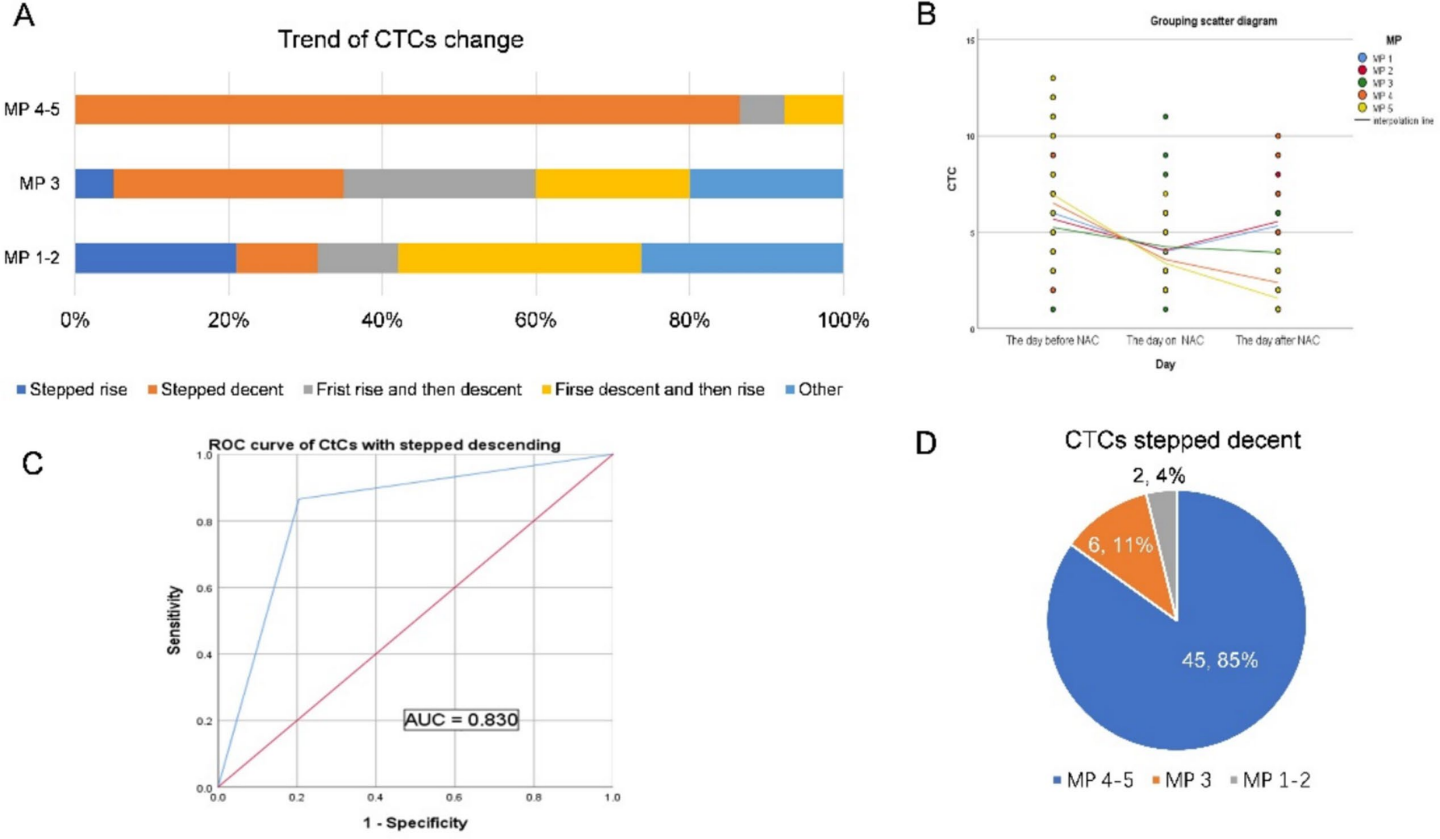
**(A,B)** The changing trends of CTCs in 91 patients who did not change the NAC regimen are shown in **(A,B)**. **(C)** Based on the ultimate chemotherapeutic efficacy of MP4-5 as the standard, the ROC curve of the stepwise decline in CTCs is shown in the figure, and the AUC is 0.830. **(D)** A total of 45 patients (85%) with stepped-down CTCs had MP4-5 as their final pathological presentation.

The changing trends of CTCs in the 91 patients who did not change the NAC regimen are shown in Figures 4A,B. Based on the ultimate chemotherapeutic efficacy of MP4-5 as the standard, the ROC curve of the stepwise declined CTCs is shown in Figure 4C, and the AUC is 0.830, with sensitivity and specificity as shown in Table 2. A total of 45 patients (85%) with stepped-down CTCs had MP4-5 as their final pathological presentation (Figure 4D). Additionally, the proportion of patients with changing trends in CTCs among patients with unchanged protocol is presented in Supplementary Figure 1.

## Discussion

All patients enrolled in this study had locally advanced triple-negative breast cancer, and compared with foreign studies, the drug resistance rate was high (27% of patients changed the neoadjuvant chemotherapy regimen due to chemotherapy resistance), while the pCR rate was low (pCR rate 17%), which was similar to other studies in China (Figure 3A) (19–21). Certain studies indicate that the survival of CTCs relies on the clustering of tumor cells, which acts as an adaptive mechanism offering physicochemical support and enhancing their stemness and growth (22). CTSCs are associated with the malignancy of breast cancer and are also associated with chemotherapy resistance and treatment failure (15, 16). As a stem cell phenotype, CD133 is mostly expressed in breast cancer with high drug resistance or malignancy. Previous studies have shown that CD133-positive tumor cells may participate in angiogenesis and enter the blood through the generated blood vessels (23). Therefore, in this study, CD133-positive CTCs were used as CTSCs and as an indicator for predicting NAC resistance in our patients. Using the ROC curve, we found that the positive expression of CTSCs on or after chemotherapy day can predict the poor efficacy of NAC in patients (progressive disease or enlarged stable disease), with specificity as high as 91%, and that the majority of patients changed their chemotherapy regimen in the third or fifth cycle (Figure 3B). NAC is a systemic therapy, and the goal of “killing” cancer cells is achieved by chemotherapeutic drugs entering the circulatory system (24). Neoadjuvant chemotherapy is a type of pressure for tumor survival, and some tumor cells undergo apoptosis and necrosis under this pressure. Other cells counteract this pressure through proliferation or stem cell differentiation (25). In breast cancer, cells expressing CD133 positivity are generally insensitive to chemotherapy (26). At the same time, previous studies have shown that the expression ratio of CTSCs in breast cancer after chemotherapy is higher than that before chemotherapy (27). CSCs are mainly responsible for therapy resistance, and CD133 is one of the most well-known and important cell surface markers of CSCs. There is growing evidence that linking CD133 to cancer resistance (28). If the tumor still expresses CD133 after one cycle of chemotherapy, it indicates that the tumor was originally resistant to the chemotherapy, or the tumor screened out cells resistant to the chemotherapy after the chemotherapy (29). In our study, CTCs positive for CD133 appeared on the day of the first cycle of chemotherapy or on the day after chemotherapy, suggesting that the cells were resistant to neoadjuvant chemotherapy and that the treatment protocol needed to be changed. Accordingly, a total of 19 patients who developed CD133-positive CTC on the day of chemotherapy and the day after chemotherapy were included in this study, among whom 11 patients changed their chemotherapy regimen (Figure 3C).

Further analysis of the change in the trend of CTC in patients with unchanged protocol revealed that there was a stepwise decline in CTC, hence effective chemotherapy (MP4 and 5) (Figures 4A,B). This stepwise decreasing trend, as observed in the ROC curve, can be used to predict the effectiveness of neoadjuvant chemotherapy (Figure 4C). The stepwise downward trend in total CTC count during the first cycle of neoadjuvant chemotherapy indicates that tumor cells with blood invasion from the primary focus are sensitive to neoadjuvant chemotherapy drugs. Multiple studies have shown that CTCs, which are tumor cells that are detached from tumor tissue and circulate in the blood, are consistent with primary tumors (18, 27, 30, 31). Therefore, these primary tumors are also sensitive to chemotherapeutic drugs. We can preliminarily conclude that the change in trend, particularly the stepwise downward decline of CTC in the first cycle of chemotherapy, can be used to predict the efficacy of neoadjuvant chemotherapy. Among the patients who did not change the neoadjuvant chemotherapy regimen, 53 patients showed a stepwise downward trend in CTC, and 45 of them (21 of them were pCR patients) achieved MP 4–5 pathology after neoadjuvant chemotherapy (Figure 4D).

Contrary to MP 4–5, in MP 1–3, the change in the trend of CTC is complex and diverse (Figure 4A). However, among the CTC change trends, except for the stepwise decline, other trends were generally few, and the number of people in each trend was relatively average, without statistical significance (Supplementary Figure 1A). In our study, although the number of patients with a continuous upward trend of CTC was small, it was mostly in the range of MP1-2, and only one patient had MP3 (Supplementary Figure 1B). The project group speculated that the CTC that first leaked into the blood from the primary focus was insensitive to chemotherapy drugs and that the primary focus might also have had drug resistance. The primary lesion with drug resistance increases in value under the stimulation of chemotherapeutic drugs, increasing the probability of blood entry to CTC. The CTC count thus shows a continuous rising trend. Therefore, the relationship between the changing trend of CTC and the efficacy of neoadjuvant chemotherapy should be studied further.

## Conclusion

The preliminary findings from this study confirmed that in locally advanced TNBC neoadjuvant chemotherapy, CTSCs were positive on the day or the day after the first cycle of chemotherapy, indicating that neoadjuvant chemotherapy was ineffective. The effectiveness of neoadjuvant chemotherapy can be predicted at least 6 weeks before evaluation by the RECIST method, which is conducive for clinicians so that they can change the treatment plan for patients with ineffective neoadjuvant therapy early enough and reduce the concomitant physical and economic effects. The stepwise decrease in ploidy CTC during the first cycle of neoadjuvant chemotherapy suggested that neoadjuvant chemotherapy was effective because most of the patients could reach a scale of MP4 or MP5, and that the treatment with this regimen could be continued. However, the correlation between other diversified CTCs and CTSCs changing patterns and the efficacy of neoadjuvant chemotherapy needs to be explored further.

## Data Availability

The original contributions presented in the study are included in the article/Supplementary material, further inquiries can be directed to the corresponding author.
