# Supplementary material for: Detection of circulating tumor cells that predicts the efficacy of neoadjuvant chemotherapy for locally advanced triple-negative breast cancer
Source: Front Med (Lausanne). 2025 Apr 30;12:1536971. doi: 10.3389/fmed.2025.1536971 (PMC12075245; doi:10.3389/fmed.2025.1536971)
Supplement: Supplementary file 1 [file Image_1.pdf]

Supplementary Material

Supplementary Figures 1

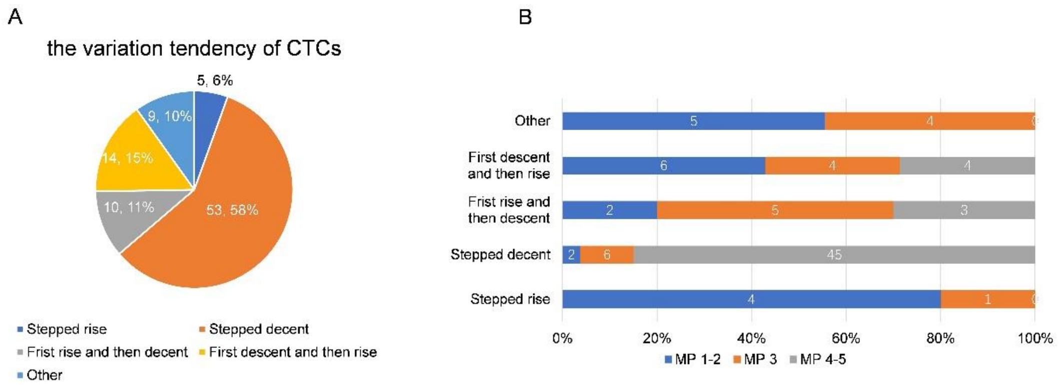

Supplementary Figure 1

(A) Among the CTC change trends, except for the stepwise decline, other trends were generally few and the number of people in each trend was relatively average, without statistical significance.

(B) The number of patients with continuous upward trend of CTC was small, but it was mostly in the range of MP1-2, and only one patient had MP3.
